# Investigating Energy-Saving Potentials in the Cloud

**DOI:** 10.3390/s140203578

**Published:** 2014-02-20

**Authors:** Da-Sheng Lee

**Affiliations:** Department of Energy and Refrigerating Air-Conditioning Engineering, National Taipei University of Technology, No. 1, Sec. 3, Chung-Hsiao E. Rd., Taipei 106, Taiwan; E-Mail: f11167@ntut.edu.tw; Tel.: +886-2-2771-2171; Fax: +886-2-2737-4919

**Keywords:** cloud sensor system, multi-dimensional data array, energy saving potentials, energy usage index (EUI), pure technical efficiency

## Abstract

Collecting webpage messages can serve as a sensor for investigating the energy-saving potential of buildings. Focusing on stores, a cloud sensor system is developed to collect data and determine their energy-saving potential. The owner of a store under investigation must register online, report the store address, area, and the customer ID number on the electric meter. The cloud sensor system automatically surveys the energy usage records by connecting to the power company website and calculating the energy use index (EUI) of the store. Other data includes the chain store check, company capital, location price, and the influence of weather conditions on the store; even the exposure frequency of store under investigation may impact the energy usage collected online. After collecting data from numerous stores, a multi-dimensional data array is constructed to determine energy-saving potential by identifying stores with similarity conditions. Similarity conditions refer to analyzed results that indicate that two stores have similar capital, business scale, weather conditions, and exposure frequency on web. Calculating the EUI difference or pure technical efficiency of stores, the energy-saving potential is determined. In this study, a real case study is performed. An 8-dimensional (8D) data array is constructed by surveying web data related to 67 stores. Then, this study investigated the savings potential of the 33 stores, using a site visit, and employed the cloud sensor system to determine the saving potential. The case study results show good agreement between the data obtained by the site visit and the cloud investigation, with errors within 4.17%. Among 33 the samples, eight stores have low saving potentials of less than 5%. The developed sensor on the cloud successfully identifies them as having low saving potential and avoids wasting money on the site visit.

## 1. Introduction

Energy saving is a crucial research topic worldwide [1–3]. Numerous studies have been conducted in Taiwan related to the energy saving potential of residential buildings under the encouragement of government policy. Their primary achievements include improving household appliances [4], high efficiency motors [5], sensor networks [6], and industrial energy-saving technology development [7]. Compared with the status existing in 2008, these studies aim to increase energy efficiency by 2% per year. Based on the energy usage baseline established in 2005, the government has stated a goal of attempting to reduce energy consumption by 20% per unit area by 2015. The carbon emissions of Taiwan by 2020 should be lowered to the amount of 2005 by employing breakthrough technologies and policies to encourage high-energy efficient equipment [8].

Before implementing energy-saving technologies and employing highly efficient equipment, the first priority is to investigate energy-saving potentials. A place with high-potential indicates low energy usage efficiency. Investing in improving energy efficiency at a place with high-potential obtains a rapid return on investment and speeds up the adoption of energy-saving technology [9]. Conversely, investing in a place with low-savings potential offers a difficult return, and improvement actions will only waste money. How to investigate energy-saving potential of buildings effectively and cost-efficiently is important for energy saving works in the future.

Several methods for determining the energy-saving potentials of buildings are discussed in published research. In 1999, Carriere *et al.* [10] developed a simulation method for evaluating the energy-saving potential of buildings. They implemented the DOE-2 simulation software, a widely used and accepted freeware building-energy analysis program, to study the energy-saving potential of large buildings. Federspiel *et al.* [11] developed and used numerical software to determine the minimum energy use intensity (EUI) for laboratories and compared this with the observed EUI of the building under investigation. The simulation method uses various factors including the properties of building construction materials, the energy efficiency of related equipment such as air conditioners and lights, and the usage periods to calculate the theoretical energy consumption of a building. By comparing with the real consumption, the energy-saving potential can be determined. Aside from simulation, statistical methods have also been developed to determine energy-saving potential. Chung *et al.* [12] used multiple regression analysis to build a benchmark table by investigating the relationship between EUIs and explanatory factors. Lee and Lee *et al.* [13] proposed adjusting the statistical method by using data envelopment analysis (DEA). The research of Lee *et al.* suggests simplified factors, including scale factors and management factors. Simplified factors could efficiently investigate the energy-saving potential of government office buildings.

Both simulation and statistical methods consider too wide a range of factors to perform a practical investigation, so any survey of energy-saving potential costs money and requires resources for collecting data. In this study, a cloud sensor system is developed for investigating energy-saving potentials. The developed system aims to fulfill the following requirements:

- Surveying energy-saving potentials only depends on information collected from webpages. This is also called a cloud sensor system.
- Surveying energy-saving potentials of distributed sites accurately.
- Identifying sites with low energy-saving potentials.

## 2. Cloud Sensor System Development

**Figure 1.**
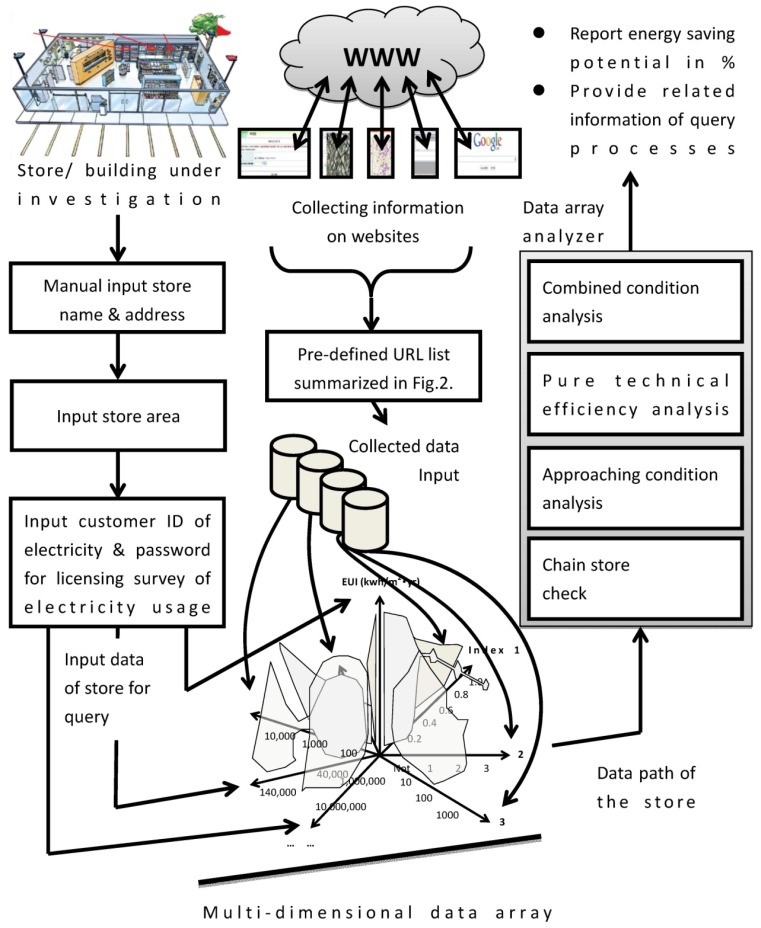
The schematic view of cloud sensor system: The left top panel and following panels show the manual input processes. From the middle top panel to down one, agent programs response for collecting data from websites with a pre-defined URL list. The pre-defined URL list will be summarized in Figure 2. It is noted that the special data architecture can be used as a sensor for investigating energy saving potentials. With the analysis algorithms shown on the right panel, the energy-saving potential is reported.

In order to meet the above requirements, structured data, an analyzer and a cloud system for initial data input are integrated in this study to develop a cloud sensor system, as shown schematically in Figure 1. As illustrated in the panels from top to bottom on the left side of the figure, the owner of a store or building under investigation registers online and sequentially inputs the store name, address, area, and customer ID of electricity. This cloud sensor system is applicable to all types of buildings, although the focus of this study is the store application. A store is a building with complex usage purposes. The primary energy consumption includes air conditioning and lighting. Certain stores are equipped with power devices such as elevators or refrigerators. People work there, pass through, stay for a while, or spend a long time shopping. The complex pedestrian flow and versatile equipment makes energy consumption and saving potential difficult to investigate. This study focused on investigating the energy-saving potential of stores that can be used for other types of buildings if the developed system works.

The panels shown in the middle of Figure 1 illustrate that the cloud system employs information collected from websites to analyze the saving potential of stores. The Uniform Resource Locator (URL) is a pre-defined list that provides the names of all websites that may contain information related to the energy consumption of stores. Using the list, data is collected to form a multi-dimensional data array, shown in the bottom panel of Figure 1. The multi-dimensional data array is a novel data structure developed in this study that consists of energy-related information of the store under investigation and other stores as a database. Referring to store input data, the cloud sensor system automatically surveys energy usage and calculates the store EUI [14]. The EUI definition in kwh/m^2^yr is expressed by:

(1) $$\text{EUI}=\frac{\text{Whole year electricity consumpion of the store}\;(\text{kwh})}{\text{Store area}\cdot \text{One year}\;(m^{2}\cdot \text{yr})}$$

The yearly electricity consumption of Equation (1) is obtained by connecting to the power company website by using an ID number. The owner inputs the store area. With these two pieces of data, the EUI value can be calculated. EUI is the benchmark of store energy usage and serves as the primary axis of the data array. All input data and collected data from websites related to the store under investigation are plotted against the EUI. The unique path across the data array can be found and input to the analyzer. Following the four-stage analysis, energy-saving potential is reported as a percentage, shown in the panels at the right side of Figure 1. The multi-dimensional data array works as the core for investigating saving potential.

To construct a multi-dimensional data array, the URL list should be specified by human experts as the pre-defined URL list. It indicates all websites that may have information related to energy consumption of the test samples.

**Figure 2.**
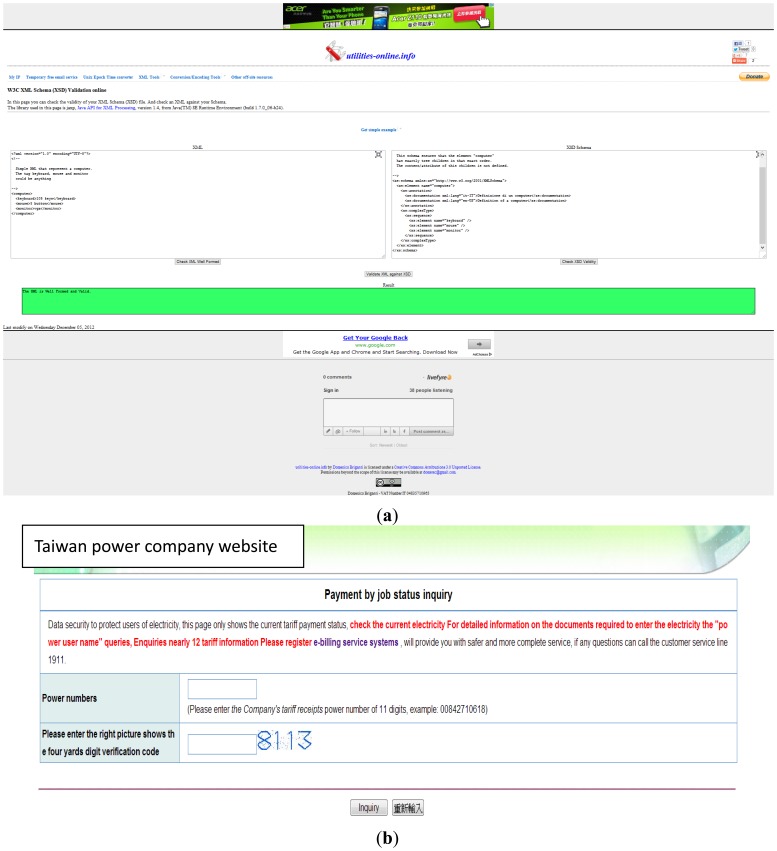

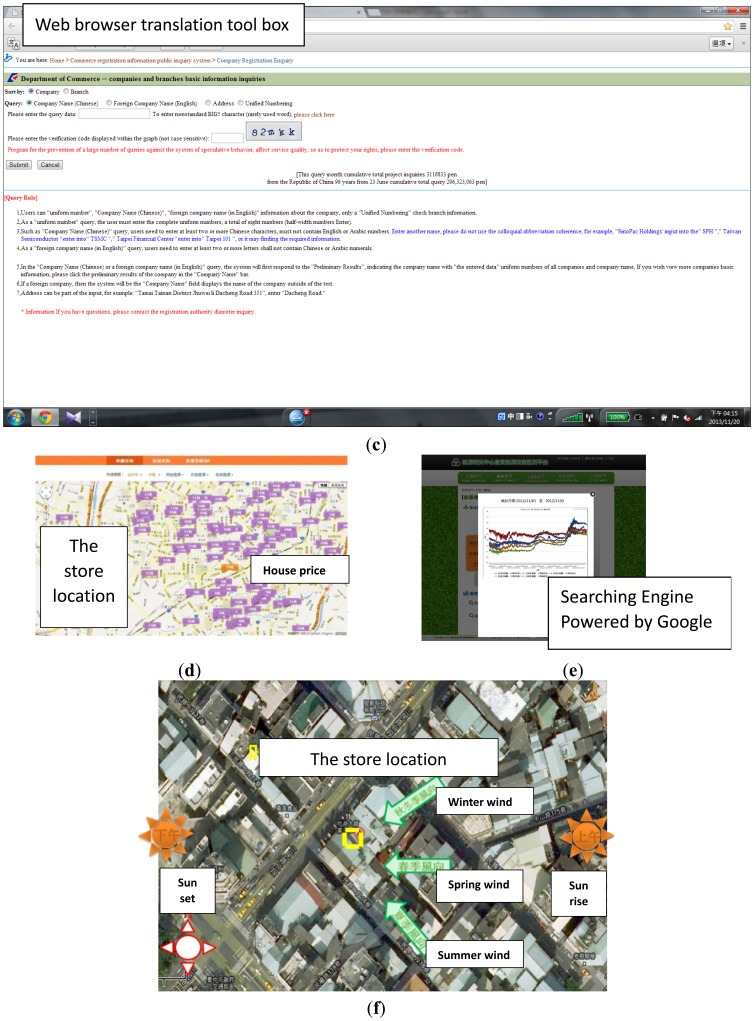
Pre-defined URL list includes: (**a**) XML program for chain store check as the first algorithm of data array analyzer; (**b**) Public website of Taiwan power company for querying energy usage of the store; (**c**) Public website of economic bureau for surveying company capital of the store; (**d**) Public website of ministry of interior for querying house price of the store; (**e**) Google website for surveying how many web pages related to the store; (**f**) Combining Google map and weather condition database, XML program can be used to estimate weather influence on energy usage of the store.

A multi-dimensional data array is the core database for the cloud sensor system developed in this study. As listed in Figure 2, all websites are related to the factors that can influence store energy consumption. A self-developed XML program, shown in Panel (a) of Figure 2, is employed to check the store name and identify whether it belongs to a chain-store series. Because the chain stores are grouped as one company, they typically use the same equipment and have similar energy consumption. Subsequently, the first priority of the pre-defined URL list is to identify the most influential factor: if the test sample belongs to a chain store.

All factors are discussed in relation to EUI. The second priority of the pre-defined URL list is to find the EUI value. By connecting to the public website of the Taiwan Power Company, the only electricity supplier in Taiwan, the energy usage of a store is surveyed, as shown in Panel (b) of Figure 2. The input data from the store owner provides the store area. As expressed by Equation (1), EUI can be calculated by the yearly power consumption counted in kWh and the store area in m^2^. With respect to EUI, the floor area of the store, one of the influencing factors on energy consumption, is discussed.

Another factor is equipment efficiency. Using a cloud-based sensor for investigating, it is difficult to ask the owner to provide all the details related to equipment. Subsequently, the pre-defined URL lists uses in a capital survey instead of an equipment investigation. By connecting to the public website of the Economic Bureau to survey the company information, the store capital can be investigated using the cloud sensor system, as shown in Panel (c) of Figure 2.

The next is to check the pedestrian flow of the store. This is a complex factor influencing energy consumption. Store owners usually can't provide, and may not know the exact pedestrian flow. In this study, the pre-defined URL list uses the public website of the Ministry of the Interior for querying the price of the store and Google for surveying the number of web pages related to the store, shown in the Panels (d) and (e) of Figure 2, respectively. The price indicates whether the store is located in a booming business area, which is directly correlated to the pedestrian flow crossing through the store. Afterward, using Google to survey the exposure frequency on web related to the store indicates how well-known the store is and its ability to attract pedestrian flow.

**Figure 3.**
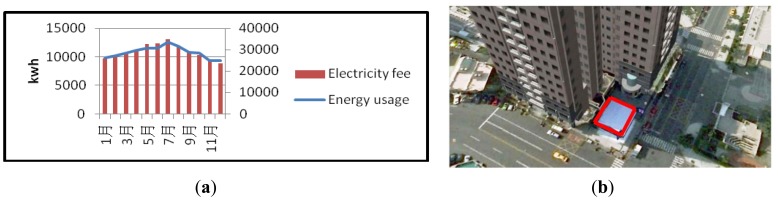
Energy usage index, EUI, is determined by surveying energy usage on the website of Taiwan power company as shown in (**a**) and the store area as shown in (**b**).

The last step is to check the influence of climate conditions. Combining the Google map and the weather condition database, a XML program is used to estimate the weather influence on the energy usage of the store. Weather influence can be divided into two factors: solar radiation shielding and wind shielding. Using the Google map, the heights of buildings neighboring the store are examined to check if they shield the store [15]. As shown on Panel (f) in Figure 2, the sun rise and set directions are known. The known heights of neighboring buildings can determine the solar-radiation shielding factor. The weather condition database can also indicate the wind direction. Panel (f) only shows the wind direction related to the store during the spring, summer, and winter. The program can survey the wind direction daily and check whether a building higher than the store shields it from wind to determine the wind-shielding factor. For details regarding solar-radiation shielding and wind shielding, please refer to Appendix. Determining height of a building by Google Earth and calculating influences of weather conditions.Using the pre-defined URLs listed in Figure 2, all factors influencing the energy consumption of the store are surveyed using the cloud. For example, the EUI is surveyed and shown in Figure 3.

As shown in Figure 3, EUI is determined by connecting to the website of the Taiwan Power Company and the store area. Using customer ID, the yearly energy consumption and electricity fee can be obtained as shown in Panel (a). The owner inputs the store area, which is confirmed using the Google map, shown in Panel (b). EUI is calculated as the primary axis of the multi-dimensional data array.

In this study, an 8-dimensional (8D) data array is constructed as a core for the proposed cloud sensor system. Data includes a check if the store belongs to a chain store series, store area, store capital, store exposure frequency on the web, price of the store and weather conditions. All factors are important for the energy usage of stores and discussed in relation to EUI. A real case study including 100 stores was performed. Details about the stores are illustrated in the following section.

## 3. Case Study—Investigating the Energy-Saving Potential of Stores on the Cloud

To verify the effectiveness of the proposed cloud sensor system, a case study was performed. The experiments include 100 stores with various business activities. Of the 100 stores 67 of them have been improved to optimal energy efficiency and were investigated as the references. The other 33 stores are not been upgraded and were treated as the test samples in this study. Stores can be divided according to four store types and area ranges from 18 to 250 m^2^. The primary energy consumption of these stores is lighting and air conditioning. Among the four store types, some must be investigated using the sensor on the cloud to determine their energy-saving potentials. All these samples are investigated and improved under the support of governmental projects. Because of privacy issues, this study cannot list the sample names, and instead serial numbers are used to encode them. All stores are listed in Table 1.

As listed in Table 1, samples are used to test the accuracy of the cloud sensor system. Data regarding the 100 stores are collected on the cloud according to the pre-defined URL list. The input data, including store name, address, area, and customer ID for surveying electricity usage are obtained from an application form completed by store owners who were willing to participate in this project, and all data are confidential. The data of 67 stores is only used to form a data array as the core of the cloud sensor system, formatted by the management number. After constructing the data array, the test is performed by surveying the energy-saving potentials of 33 stores that are unimproved to optimal energy efficiency. To verify accuracy, the research team visited the 33 stores to help them improve energy efficiency by using on-site measurements to obtain the real energy potential of each store. Accuracy is confirmed by comparing the saving potential obtained by the site visit and that obtained by the cloud investigation.

## 4. Constructing an 8D Data Array as a Sensor Core to Investigate Energy Saving Potentials

**Figure 4.**
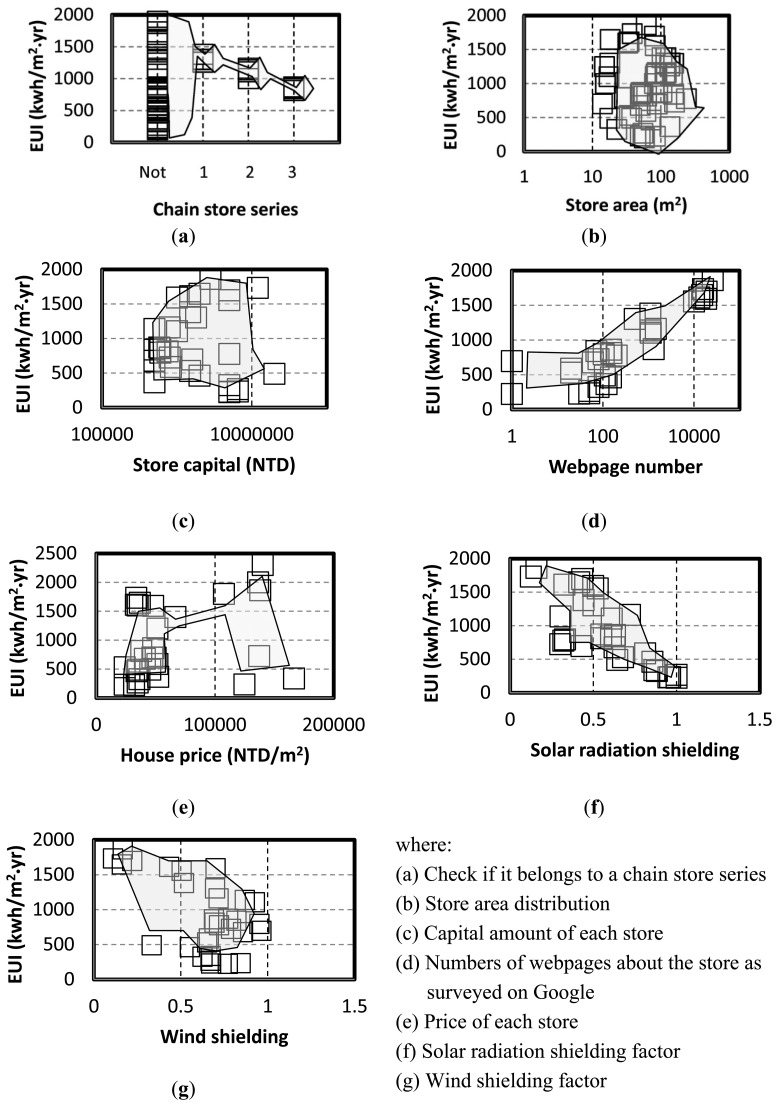
All data collection from 67 stores according to pre-defined URL list and data envelop lines.

With data from stores of case study as discussed in previous section, an 8D data array is constructed to determine energy-saving potentials. The pre-defined URL list provides all the website connections used to obtain related information for investigating the energy-saving potential by using the cloud. All gathered information, including the store ownership, store area, store capital, number of webpages related to the store surveyed on Google by using the keyword of store name *etc.*, and the price of the store, is plotted according to the EUI. After collecting 67 stores as references, the data points and envelope lines are plotted, as shown in Figure 4.

**Figure 5.**
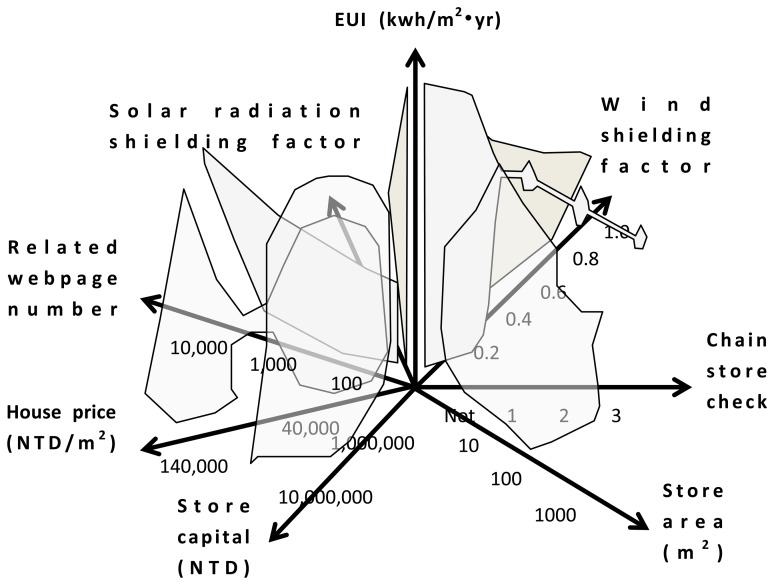
With EUI and collected data summarized in Figure 4, an eight-dimensional (8D) data array is constructed as a sensor core to investigate energy-saving potential on the cloud.

The envelope line is fundamental to DEA [13] and consists of the data point boundary. Figure 4 shows the data points by envelope line to investigate energy-saving potentials. Panel (a) in Figure 4 shows that the EUI values of chain stores have a certain range regarding different series, similar to the conclusion of the authors' previous study [16], which requires checking if a store belongs to a chain-store series. Otherwise, stores have distributed EUI values, shown on the left, and must be investigated using other information collected from websites. Panels (b) and (c) show that the EUI values have a uniform random distribution of store area and capital. Each distribution shows the shape has the widest dimension across the EUI axis. From the widest scale, the most common area and capital of the surveyed store samples can be identified, which is correlated to two influencing factors: the floor area and the equipment efficiency of the store. Panel (d) provides the number of webpages obtained by the keyword survey on Google. After deleting the pages with duplicate content, the number indicates how many discussions relate to the store, which is a proxy index of pedestrian flow. Another index is the price shown in Panel (e), which indicates the volume of pedestrian flow passing through the business district where the store is located. Combining the data from Panels (d) and (e) identifies the influencing factor of pedestrian flow. Panels (f) and (g) provide the solar-radiation shielding and wind-shielding factors, illustrating the last influencing factor on energy consumption: climate conditions. These data collections and envelop lines are used to construct an 8D data array, the core of the cloud sensor system for investigating the energy-saving potential on cloud. Plotting all data regarding the EUI enables construction of an 8D data array as a sensor core for investigating the energy-saving potential on the cloud, shown in Figure 5.

**Figure 6.**
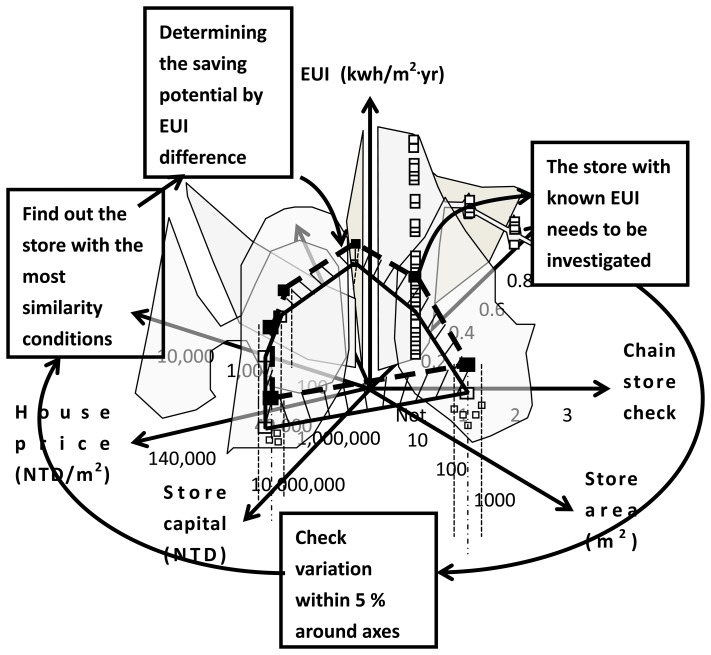
Similarity condition analysis as the second algorithm data array analyzer: data array is used to find out a reference which has the most conditions similar to the store under investigation. EUI comparison between sample and reference is the basic principal for investigating energy-saving potential on the cloud. Four blocks around the data array illustrate how to determine saving potential.

The main working principal of the data array is searching and finding the information correlation of the investigated topic. This study investigates the energy-saving potential of stores by constructing an 8D data array. The data collection of the 67 stores illustrated in Table 1 is used as the reference to construct the data array. Based on the pre-defined URL list as discussed in Figure 1, the cloud sensor system surveys the EUI of the store and other information including the chain store check, the store area, store, capital, *etc.* With a known EUI, the data array checks for a reference with the most similar conditions to the 33 stores that are unimproved to optimal energy efficiency. The similarity condition is defined as within 5% variation related to the data point at each axis. For example, the store area variation between the reference and the test sample is less than 5% of the area of reference. Along the seven axes, the one that satisfies the 5% variation rule and has the lowest variation is the selected reference. Comparing the EUI difference between the reference and the store under investigation determines the energy-saving potential. The algorithm is shown in Figure 6.

As illustrated in the caption of Figure 6, finding a reference sample with the most similarity conditions is the fundamental analysis method. The similarity condition analysis denoted in the block of the data array analyzer as part of the cloud sensor system shown in Figure 1 provides a basic method to determine energy-saving potential. However, for not all 33 stores under investigation is it possible to find a reference sample within 5% variation around the axes of the data array. For these cases, pure technical efficiency is used instead of the similarity condition analysis for choosing the reference [13,17].

**Figure 7.**
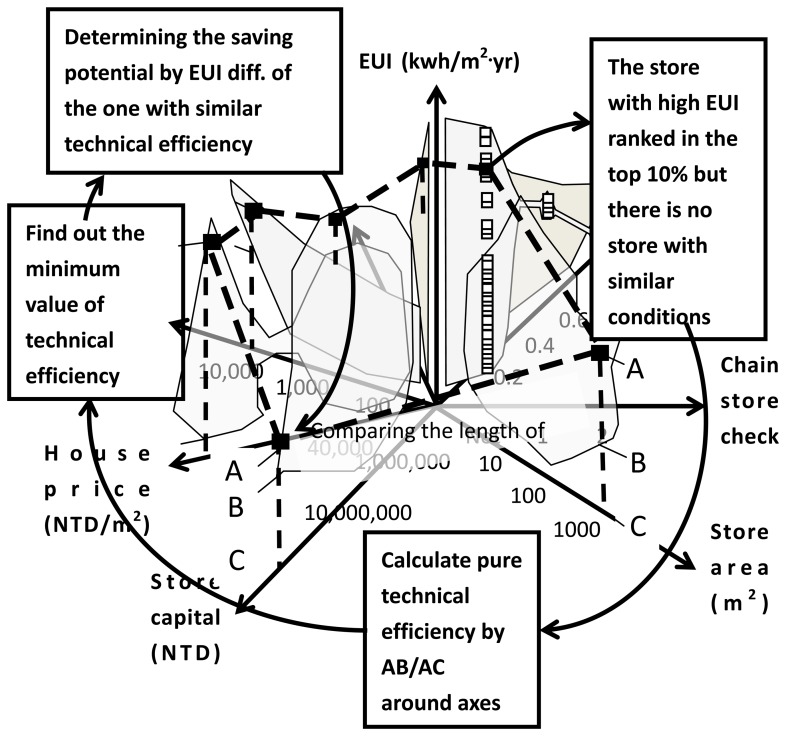
Pure technical efficiency analysis as the third algorithm data array analyzer: some stores with high energy usage may have no store with similar conditions. Pure technical efficiency is used to determine energy-saving potential from the data array by comparing EUI difference of the one with the most similar efficiency.

Figure 7 shows how to determine pure technical efficiency to determine the reference sample. The store with a high EUI ranking in the top 10% of samples on the data array typically cannot determine the store with a similarity condition. This study uses pure technical efficiency to determine energy-saving potential on the data array by comparing the EUI difference of the sample with the one has the most similarity conditions.

Figure 7 shows that certain stores having high EUI value cannot determine a reference with similar conditions. Pure technical efficiency is used to indicate energy-saving potential. Based on the DEA discussed in Section 2, the envelope line instead of data points is used to indicate the EUI along axes. Although there is no similarity point, the envelope line always exists. Saving potential can be determined by:

(2) $$\text{Pure technical efficiency}=\frac{\bar{\text{AB}}}{\bar{\text{AC}}}$$

where AB indicates the EUI value of the store under investigation and AC provides the EUI value on the lower limit of the envelope line. Points A, B, and C can be referred to in Figure 7. The analysis primarily relies on the scale length. The scale provides the saving potential in theory, or pure technical efficiency. Along the seven axes, the efficiencies are checked to find the minimum value. For a store with high energy consumption for which we cannot find a similar case on the data array, the saving potential is determined by the EUI difference at the axis with minimum technical efficiency.

**Figure 8.**
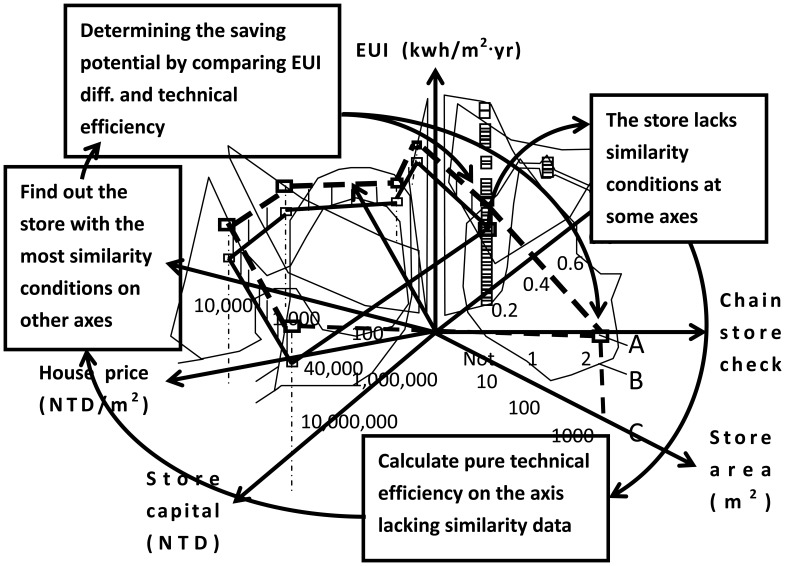
Combined condition analysis as the fourth algorithm data array analyzer: some stores may lack of similarity conditions at some axes of data array. Technical efficiency and similarity conditions are combined to determine energy saving potential.

Pure technical efficiency is also employed to analyze the case that lacks similarity conditions on certain axes. Figure 8 shows the analysis of the combined conditions. As shown in Figure 8, for a lack of similarity conditions on certain axes one can use pure technical efficiency for the analysis. A lack of similar conditions on certain axes causes a combined condition. Energy-saving potential is determined by the minimum EUI differences related to the similarity conditions and pure technical efficiency.

## 5. Experiment in Contrast—EUI Rating Method

In order to check if the cloud sensor system developed in this study has any advantages over existing techniques, a comparison experiment was performed. Referring to the website of Oak Ridge National Laboratory [18], energy use and cost reductions can be estimated for building EUI ratings. This website function is the same as the cloud sensor system developed in this study. Subsequently, the experiment used this website to investigate energy-saving potentials, *i.e.*, cost reductions of 33 sample stores, and compared with the results obtained from cloud sensor system as a contrast.

**Figure 9.**
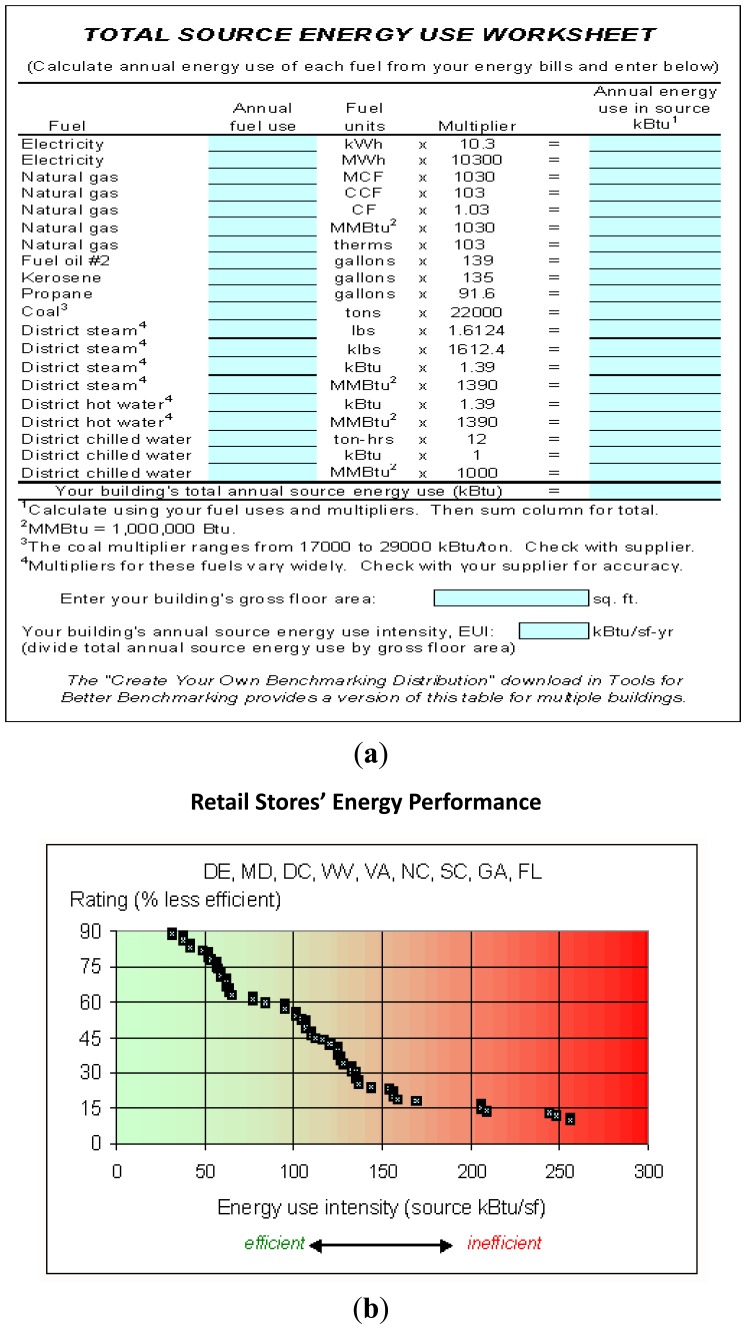
EUI rating method in the comparison experiment [18]: Oak Ridge National Laboratory promoted the method to estimate energy-saving potentials by building EUI rating. EUI value is obtained by input data of energy usage as shown in (**a**); using retail stores' energy performance as shown in (**b**), energy use and cost reductions can be estimated.

The EUI rating method relies on input of energy usage data online. To rate EUI, the laboratory uses the data plots collected from cumulative histograms of U.S. regions. In this study, retail stores' energy performance in the state of Florida (FL) is used as the data plot since weather conditions of this state are the most similar to Taiwan. Then, to locate the total energy use intensity of 33 sample stores on the x-axis of data plot, we move up vertically to the curve and then left to the y-axis to get EUI rating—the percent of similar buildings in the U.S. less efficient than the sample store. The procedures of the EUI rating method are shown in Figure 9.

The lower the percentage rating, the higher savings potential for energy use and cost reductions. If a sample store ranks below 20%, there are often large energy cost savings available through improved energy management or equipment upgrades. Substantial opportunities are also often achievable for buildings rated between 20% and 50%. For ratingd above 60%, the energy-saving potential could be less than 20%. The EUI rating method uses store ratings to determine saving potentials as shown in Table 2.

Though there are many researches related to estimate energy-saving potentials [10–13,19–25], there are few studies which can provide a survey of energy-saving potentials only depending on information collected from webpages. Only the website promoted by Oak Ridge National Laboratory has a similar concept to the cloud sensor system developed in this study. Subsequently, this study refers to this website, gets the results for the 33 stores under investigation and compares the related results. The most important is to test the function of the 8D data array and compare its ability with the EUI rating method, the methodology promoted by Oak Ridge National Laboratory.

## 6. Results and Discussion

The proposed sensor on the cloud to investigate stores only depends on information collected from webpages. That fulfills the first requirement as discussed in the Introduction section. The case study includes 100 stores to test the cloud sensor system. Among them, 33 stores are unimproved to optimal energy efficiency. This research used the cloud sensor system and the 8D data array to investigate energy-saving potential and compared it with the data obtained from the EUI rating method promoted by Oak Ridge National Laboratory and those obtained during a site visit. The EUI rating method provides a contrast to the system developed in this study. The comparison results were used to evaluate if the developed system fulfills the accuracy requirement. Aside from that, whether the system can identify stores with low energy saving potentials of less than 5% is another check point.

**Figure 10.**
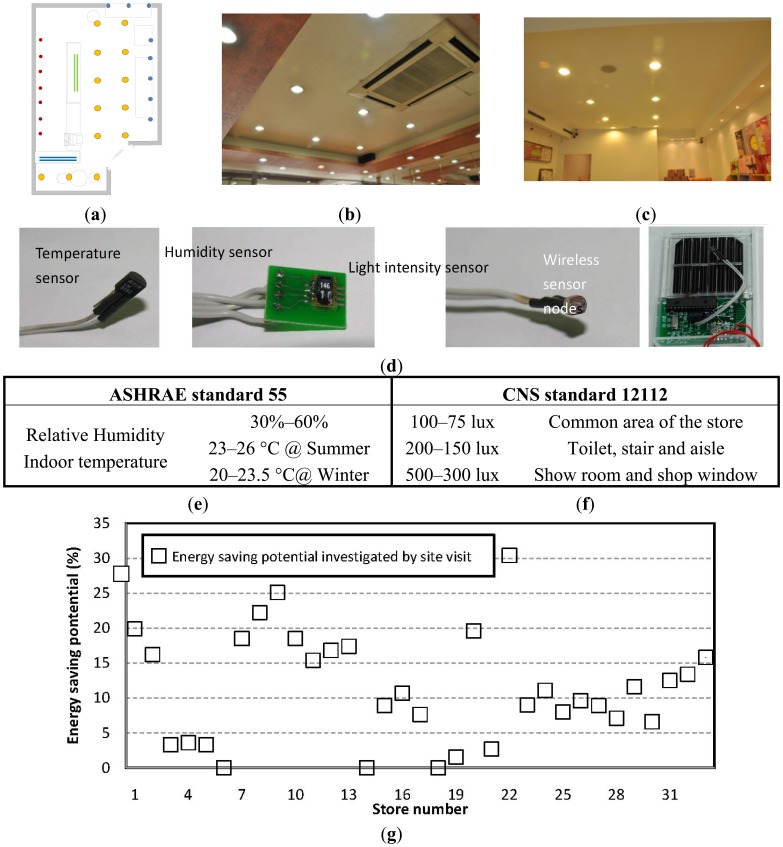
Site visit to investigate energy saving potential of store: (**a**) Plot floor plan and check equipment includes; (**b**) air conditioner; (**c**) lighting system; Using sensors and wireless nodes to survey environment (**d**); Adjust air conditioning setting and light intensity according to ASHRAE standard (**e**) and CNS standard (**f**); Get energy saving potential of each store (**g**).

The energy-saving potential investigation according to the site visit is briefly illustrated in Figure 10. Researchers visited the store, plotted the geometry, and denoted the main equipment in the store, as shown in Panel (a) of Figure 10. For this case, the primary energy consumption sources of stores are air conditioning and lighting. Thus the air conditioner type and arrangement of the artificial lighting system were investigated in detail, as shown in Panels (b) and (c) of Figure 10. Sensors, including a temperature sensor, humidity sensor, light-intensity sensor, and a wireless node module were distributed around the store to collect environmental data, shown in Panel (d). According to ASHREA standard 55, the air conditioner setting was adjusted to a thermal comfort level with a temperature and humidity range, illustrated in Panel (e). The light bulb number was also reduced to a suitable indoor light intensity according to the CNS (Chinese National Standard) 12112, briefly summarized in Panel (f). Through an optimized air conditioner setting and a suitable light-intensity adjustment, energy consumptions before and after were evaluated to obtain saving potentials. Panel (g) plots the energy-saving potential of the 33 stores.

Table 3 presents a summary of the detailed energy-saving potential data and briefly lists the primary methodologies for improving energy efficiency. The maximal savings potential in an eyeglass and accessories store reached 30.4%. The primary reason for energy waste was over-lighting and overly cold air conditioning. By reducing artificial light intensity, changing the high efficiency lighting system, and adjusting the air conditioner temperature, the store effectively improved energy efficiency and quickly earned back its improvement investment. However, among the 33 stores, eight stores had a saving potential of less than 5%. These stores do not need to invest in improvement or to be investigated in a site visit. In this study, 33 stores were investigated according to the developed cloud sensor system. Collecting 1-year data regarding power consumption from the Taiwan Power Company website determines the EUI of each store. From the pre-defined URL list, information on websites related to these stores was collected and input to the data array constructed by the data of the other 67 stores that had been improved to optimal energy efficiency to generate a data file for each store. Although the sensor on the cloud collects data from two XML programs and five website connections, all data on a store can be collected in 35 s on average. The data array analyzer analyzes the data according to the EUI difference and pure technical efficiency. All computations involve only sums and divisions. The simple algorithm from the comparison and DEA makes the cloud-computing easy to use and does not require large computing resources. The energy-saving potential report of a store can be generated within a short time of less than several minutes. The cost approaches zero if not accounting for manpower.

Determining the optimal air conditioner setting and how to adjust the lighting requires a monitoring period of 2 days or more during the summer and winter. The cost of a site visit to each store differs according to the store area. The average store investment is approximately 15,000 NTD. Considering the time and cost, the cloud sensor system provides an efficient way to investigate the energy-saving potentials of stores.

**Figure 11.**
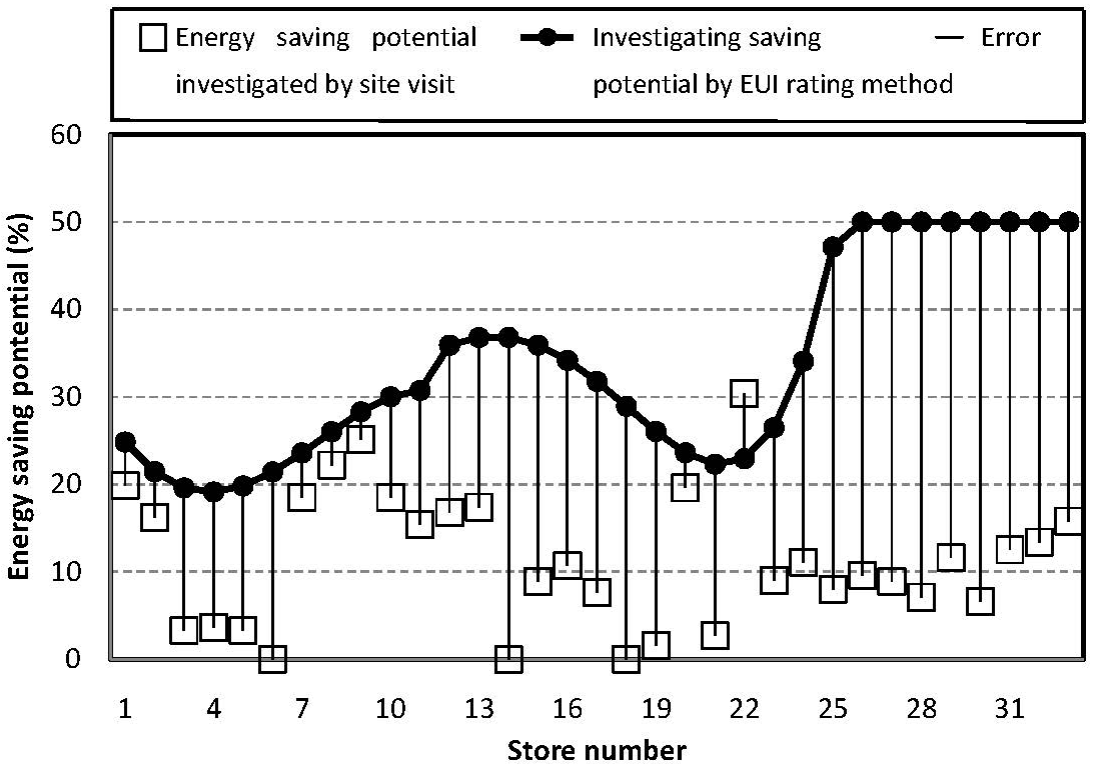
EUI rating method is employed to investigate energy-saving potential. Investigation gives errors around 22.7%. There are eight stores that have low saving potentials of less than 5%. The EUI rating method can't identify them.

Despite its high efficiency, the sensor on the cloud needs to prove its accuracy. Aside from the developed system, Oak Ridge National Laboratory has also promoted a EUI rating method to estimate energy use and cost reductions by data collection on websites. The function is almost the same as the developed system, but s different algorithm is promoted to determine energy-saving potentials of stores. Results obtained from our case study not only need to prove the accuracy of the developed system but also have to prove it's better than the EUI rating method. The energy-saving potential of the 33 stores obtained from EUI rating method is reported in percentages and plotted in Figure 11. The energy-saving potential obtained from site visits is also plotted for reference. By comparing the results of the cloud investigation and the site visit, the trend and errors are discussed.

As shown in Figure 11, the errors between the EUI rating method and the site visit are around 22.7%. Most results are overestimated and the investigation trend doesn't match the savings potential obtained by the site visit. Eight stores having a low savings potential less than 5% can't be screened out. The EUI rating method and data collection on websites can't be effectively used to estimate energy use and cost reductions of Taiwan stores. That may be due to different weather conditions and business status. Through collecting more information including the chain store check, company capital, price, and the influence of weather conditions on the store; even the exposure frequency of store under investigation, the cloud sensor system is expected to provide more accurate estimations of energy-saving potentials.

**Figure 12.**
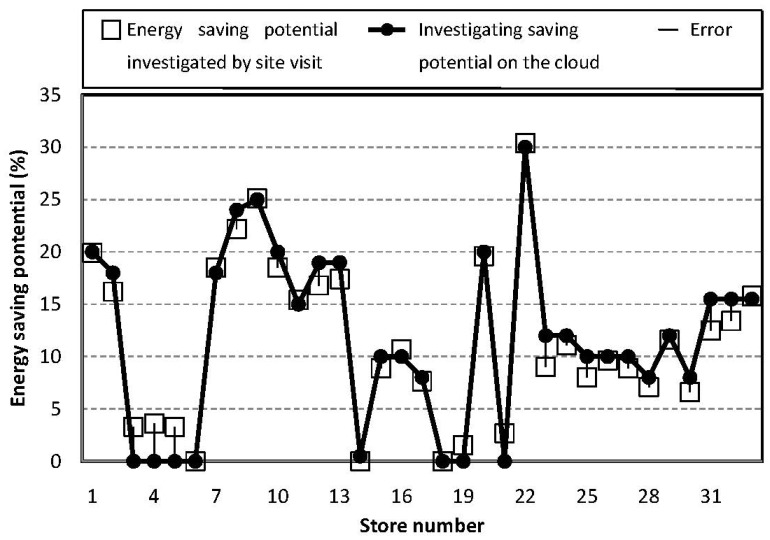
Cloud sensor system uses 8D data array to investigate energy saving potential. Investigation gives errors within 4.17%. It's noted that the trend of investigation fully matches the real saving potential obtained from site visit and eight stores have low saving potential less than 5% are successfully screened out.

The energy-saving potential of the 33 stores obtained from the cloud sensor system is reported in percentages and plotted in Figure 12.

As shown in Figure 10, the errors between the cloud investigation and the site visit are within 4.17%, and the investigation trend fully matches the savings potential obtained by site visits. Eight stores having a low savings potential of less than 5% were screened, indicating that the cloud-computing system effectively identifies those without potential for improvement, saving on the cost of the site visit and improvement efforts.

Comparing the results of Figures 11 and 12, the comparison experiment illustrates that the cloud sensor system developed in this study has better performance for estimating energy-saving potential of Taiwan stores then the existing EUI rating website. In addition, the results also illustrate that the cloud sensor system satisfies all requirements promoted in the Introduction section. Good agreement between the data obtained by the site visit and the cloud investigation proves the accuracy of the cloud sensor system. Among the 33 samples, eight stores have low energy-saving potentials of less than 5%. The developed sensor on the cloud successfully detects the ones with low saving potential and avoids wasting money on site visits.

## 7. Conclusions

The cloud-computing system developed in this study provides an efficient tool for investigating the energy-saving potential by using the cloud. The following three conclusions are made:

- The cloud sensor system efficiently and economically surveys the energy-saving potential of sites. A site-visit investigation takes 2 days or more to complete a store report. The cloud- computing system only takes 35 s on average. A site visit costs 15,000 NTD on average for one store. A cloud investigation only requires a communication fee less than 0.1 NTD, saving substantial work time and costs.
- Compared with the data obtained by a site visit, the cloud sensor system reports energy-saving potentials with accuracy of less than 5%. For most physical sensors, the accuracy is within this level. Using the structured data, an 8D data array, a cloud sensor is proven to have the same accuracy as a real sensor.
- Web searching has become a popular human behavior. Search engines, such as Google, list all information correlated to search keywords. In this study, a multi-dimensional data array and special geometry provided more accurate information than searching does. The cloud sensor works like a real sensor. The application may not be limited to surveying energy-saving potential, and can expand to other sensing environments, detecting pedestrian flow, traffic flow, and other promising applications.

## Figures and Tables

**Table 1.** Store list of the case study: Experiments have 100 samples in total. Among them, 67 stores have been improved to optimize their energy efficiency and 33 stores need to be investigated and improved.

**No.**	**Store Type**	**Area (m^2^)**	**Note**
1–17	Eyeglasses and auxiliaries store	29.3–158.7	Four of them need to be investigated and improved to better energy efficiency. Others have been improved to optimized energy efficiency.
18–37	Clothes store	18–250	Two of them need to be investigated and improved.
38–70	Grocery store and convenience store	80.6–236.7	Fourteen of them need to be investigated and improved.
71–100	Bakery and food store	22.74–62	Thirteen of them need to be investigated and improved.

**Table 2.** EUI rating method using store ratings to determine saving potentials.

**Rating for Store**	**Energy Use and Cost Reduction Potential (%)**
below 20%	above 50%
20% to 40%	35% to 50%
40% to 60%	20% to 35%
above 60%	below 20%

**Table 3.** In order to verify the effectiveness of investigating energy saving potential on cloud and check, 33 stores are investigated by site visit. Energy saving potential is listed below.

**No.**	**Store Type**	**Energy Saving Potential**	**Main Improving Methodology**
1–4	Eyeglasses and accessories store	8%–30.4%	Reduce artificial light intensity, change high efficiency lighting system and Control air conditioner temperature
5–6	Clothes and accessories store	18.5%–21.5%	Control air conditioner temperature and reduce leak of cooling air
7–20	Grocery store and convenience store	0%–13.4%	Change air conditioner with high energy efficiency
21–33	Bakery and food store	0%–19.6%	Reduce artificial light intensity and change high efficiency lighting system

Summary		Only one store has high saving potential around 30% and needs to be improved soon due to the high return rate of investment. Eight stores have potential less than 5% and there's no need to investigate them by site visit. Investigating on the cloud must screen out these stores to avoid money waste.	

**Figure A1.**
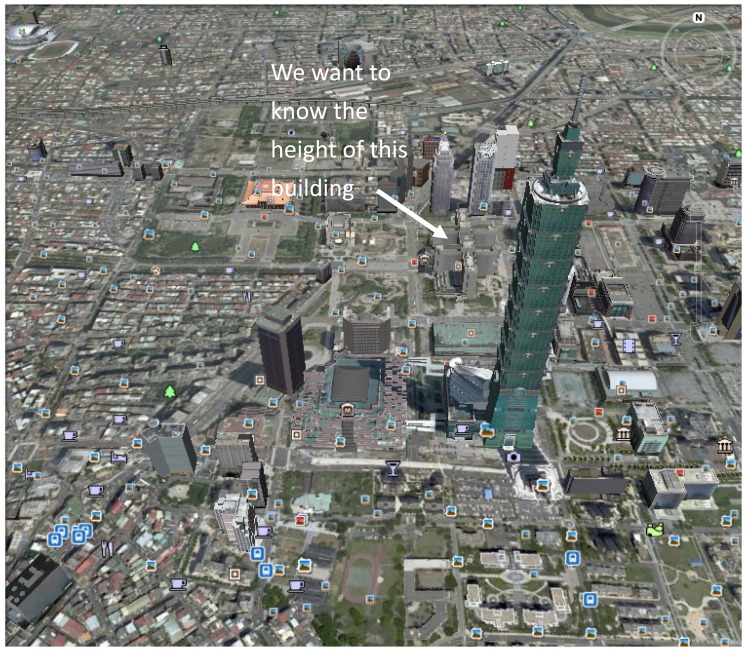
Open Google Earth for locating the building.

**Figure A2.**
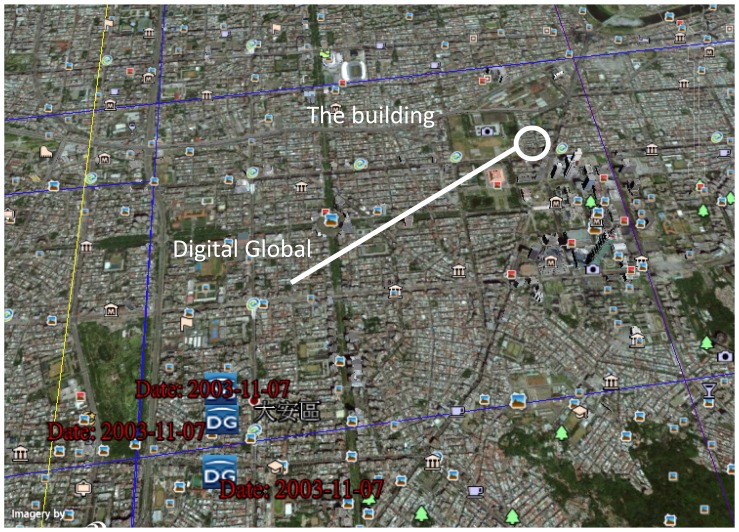
Find out Digital Global index.

**Figure A3.**
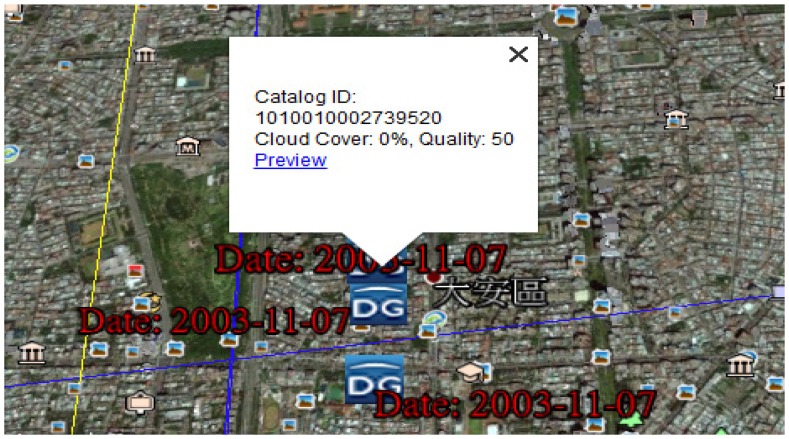
Open Preview to get the Avg Off Nadir Angle.

**Figure A4.**
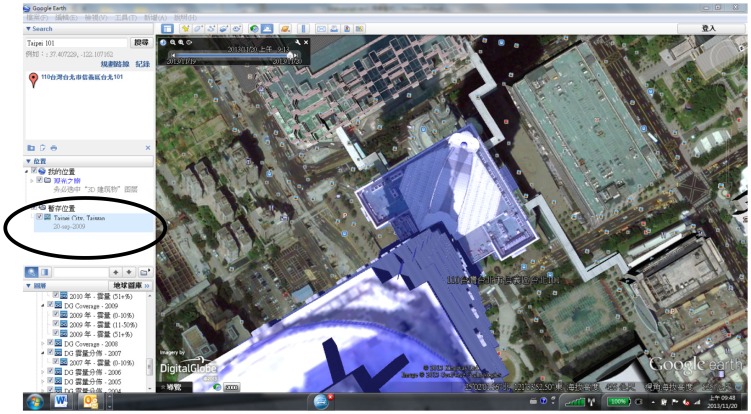
Tool box window of Digital Global view for observation of the building.

**Figure A5.**
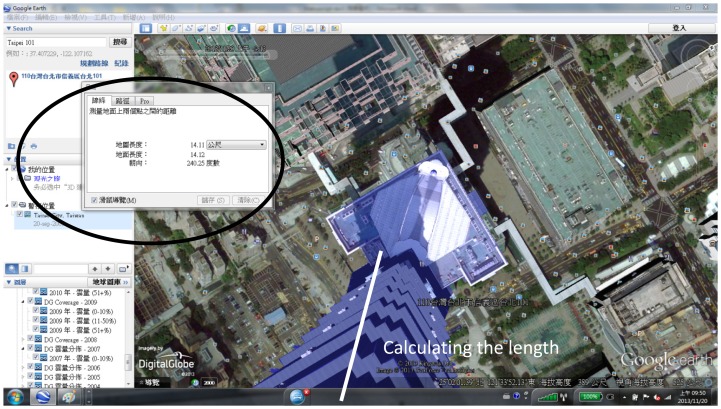
Using tool box to calculate length on Digital Global view.

**Figure A6.**
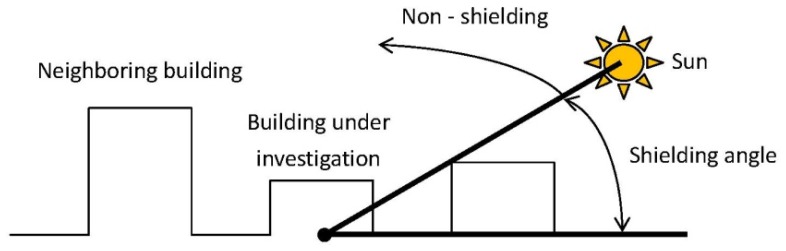
Schematic view of determination of building and neighboring buildings.

## Appendix

### Determining Height of a Building using Google Earth and Calculating the Influences of Weather Conditions

How to determine height of a building by Google Earth mainly relies on calculating the “Avg Off Nadir Angle”. The procedures are as follows:

- Open Google Earth to locate the building under investigation (Figure A1).
- Check which Digital Global is near the building (Figure A2).
- Use the preview function of Digital Global to get the “Avg Off Nadir Angle” (Figure A3).

Using Digital Global view to observe the building (Figure A4).

- Using rule function to get the length on Digital Global view (Figure A5).
- Calculate the height of the building by:
  Length on Global View/tan(Avg Off Nadir Angle) = Height of building under investigation.
- With the height of building and neighboring buildings, solar radiation shielding can be estimated as follows (Figure A6):
- Calculate solar radiation shielding factor by:
  Whole day shielding angle/180 = Solar radiation shielding factor.
- Get weather conditions from the website of the Central Weather Bureau, especially the wind direction shown in gray background (Table A1).
- Combining information related to height of buildings and wind direction, wind shielding factor can be calculated by:
  ∑Delta(Month)/12 = wind shielding factor
  where Delta(Month) is defined by
  If height of neighboring building > height of building under investigation along wind direction at the Month
  Then Delta = 1
  Else Delta = 0
  End if

**Table A1.** Weather conditions information list.

**Month**	**Temp (**°**C)**		**R.H. (**%**)**		**Wind**		**Solar Radiation (hours)**	

	Last year	Avg.	Last year	Avg.	Vel. (m/s)	Direction	Last year	Avg.
3	19.7	19.6	78	76.6	13.6	East	165.7	149.9
4	24.6	23.1	75	77.3	13.9	East	88.7	137.8
5	26.9	26.0	73	77.1	14.6	East	170.3	158.7
Spring	23.7	22.9	75.3	77	14.03	East	141.57	148.8
6	27.8	27.6	78	77.9	17.7	East	134.1	160.1
7	29.1	28.6	73	76.6	13.2	SSE	204.2	199.6
8	28.0	28.3	77	77.6	22.0	SSE	96.4	178.7
Summer	28.3	28.2	76	77.4	17.63	SSE	144.9	179.5
9	27.6	27.4	69	75.8	20.5	ENE	190.1	175.8
10	24.9	25.2	64	72.6	13.4	ENE	222.9	188.6
11	22.3	21.9	71	72.7	10.3	ENE	132.6	130.1
Fall	24.9	24.8	68	73.7	14.73	ENE	181.87	186.3
12	18.4	18.1	73	72.3	13.5	ENE	143.6	182.3
1	16.9	16.6	74	74.6	14.7	ENE	184.2	176.6
2	19.4	17.3	74	76.8	13.1	East	174.7	140.6
Winter	18.2	17.3	73,7	74.6	13.77	ENE	167.5	166.5
